# High temperature *in-situ* synchrotron-based XRD study on the crystal structure evolution of C/C composite impregnated by FLiNaK molten salt

**DOI:** 10.1038/s41598-017-11033-2

**Published:** 2017-09-06

**Authors:** Shanglei Feng, Yingguo Yang, Li Li, Dongsheng Zhang, Xinmei Yang, Huihao Xia, Long Yan, Derek K. L. Tsang, Ping Huai, Xingtai Zhou

**Affiliations:** 1 Shanghai Institute of Applied Physics, Chinese Academy of Sciences, 2019 Jialuo Road, Shanghai, 201800 China; 2 Shanghai Synchrotron Radiation Facility, Shanghai Institute of Applied Physics, Chinese Academy of Sciences, 239 Zhangheng Road, Shanghai, 201204 China; 3 University of Chinese Academy of Sciences, Beijing, 100049 China

## Abstract

An *in-situ* real-time synchrotron-based grazing incidence X-ray diffraction was systematically used to investigate the crystal structural evolution of carbon fiber reinforced carbon matrix (C/C) composite impregnated with FLiNaK molten salt during the heat-treatment process. It was found that the crystallographic thermal expansion and contraction rate of interlayer spacing *d*_002_ in C/C composite with FLiNaK salt impregnation is smaller than that in the virgin sample, indicating the suppression on interlayer spacing from FLiNaK salt impregnated. Meanwhile the crystallite size *L*_C002_ of C/C composite with FLiNaK salt impregnation is larger than the virgin one after whole heat treatment process, indicating that FLiNaK salt impregnation could facilitate the crystallization of C/C composite after heat treatment process. This improved crystallization in C/C composite with FLiNaK salt impregnation suggests the synthetic action of the salt squeeze effect on crooked carbon layer and the release of internal residual stress after heating-cooling process. Thus, the present study not only contribute to reveal the interaction mechanism between C/C composite and FLiNaK salt in high temperature environment, but also promote the design of safer and more reliable C/C composite materials for the next generation molten salt reactor.

## Introduction

Molten salt reactor (MSR) has been regarded as one of the most promising candidates for Generation IV Nuclear Energy System because of its unique fuel cycle capabilities and safety characteristics^1–3^. Control rod guider plays an important role in a reactor, as the guider is used to protect the control rods which could effectively control the nuclear chain reaction^4^. Carbon fiber reinforced carbon matrix (C/C) composite is considered as a very good candidate material of control rod guider since C/C has a series of advantages, such as low atomic number constituents, low density, low coefficient of thermal expansion, high specific strength, high thermal conductivity, and excellent corrosion resistance^5–7^. Furthermore, the research and development on the application of C/C composite to High Temperature Gas-cooled Reactor and Very High Temperature Reactor have been carried out since 1990s, Japan Atomic Energy Agency and Toyo Tanso have carried out the Research and Development (R & D) program on C/C composite to be used for control rod^8, 9^. If C/C composite is used as control rod guider in the core of MSR, it would directly contact molten salt. Due to the porous nature of C/C composite^10^, just like nuclear graphite, molten salts are easily infiltrated into the bulk of C/C composite^11^, which would affect the properties and structure of the C/C composite, such as coefficient of thermal expansion, irradiation resistance and microstructure of the C/C composite in a particular circumstance^12–14^. Considering the safety and reliable of materials used in MSR, it is essential to study and understand impregnation mechanism of molten salts into the bulk of the C/C composite. However, little attention has been paid to the impregnation mechanism of molten salt into C/C composite, especially the properties change of the C/C composite during the infiltration process.

Recently, we have employed synchrotron-based near-edge X-ray absorption fine structure spectroscopy (NEXAFS) to investigate the interaction between nuclear graphite and molten fluoride salts^15^. Results indicate that C-F bonds are formed in the nuclear graphite after the static molten FLiNaK salt experiment^15^. We also have found the evidence for the formation of C-F bond in the carbon material and the defects in the pyrolytic carbon which facilitate the interaction between pyrolytic carbon and molten fluoride salt^16^. Similarly, a series of previous investigation results show that the impregnated phase (such as glass, metal or salt) can greatly affect the physical properties of graphite phase^17–20^. However, the microstructure evolution of C/C composite during the infiltration process should be different from that of graphite, because that a C/C composite is a unique all-carbon composite with carbon fibers embedded in a pyrolytic carbon matrix^21^. The fabrication of C/C composite is mainly composed of producing the fiber precursor and then densifying this with the carbon matrix^21–26^. Besides, all these previous studies were carried out at room temperature, which means that the results are just concentrated on the interaction between molten salts in solid state and material. To the best of our knowledge, there is no reported research about the impact mechanism of molten salt induced performance changes of C/C composite, especially in high temperature liquid molten salt. Therefore, it is highly desirable to *in-situ* study the crystal structure evolution of the C/C composite impregnated by high temperature liquid molten salt to reveal the interaction mechanism between C/C composite and FLiNaK (46.5%LiF-11.5%NaF-42%KF (mol%)) salt, since the C/C composite control rod guider in a MSR is exposed directly in the high temperature liquid molten salt.

Herein, an *in-situ* real-time synchrotron-based grazing incidence X-ray diffraction (GIXRD) was firstly used to determine the crystal structural evolution of C/C composite with and without FLiNaK molten salt impregnation during heat-treatment process. One dimensional GIXRD (1D-GIXRD) and two dimensional GIXRD (2D-GIXRD) patterns were obtained simultaneously to reveal the crystalline and texture changes induced by the impregnated FLiNaK salt network in graphite matrix of C/C composite. The evolutions of graphite interlayer spacing *d*_002_ and corresponding crystallite size *L*_C002_ during the heat-treatment process of C/C composite samples with and without FLiNaK salt impregnation were obtained by 1D-GIXRD. 2D-GIXRD was further investigated the crystallographic orientation of C/C composite to perform the change of crystal texture. All these achievements will contribute to deeply understand the interaction mechanism between C/C composite and FLiNaK salt in high temperature environment and promote to design the safer and more reliable molten salt reactor of next generation.

## Results and Discussion

### Morphologies

**Figure 1:**
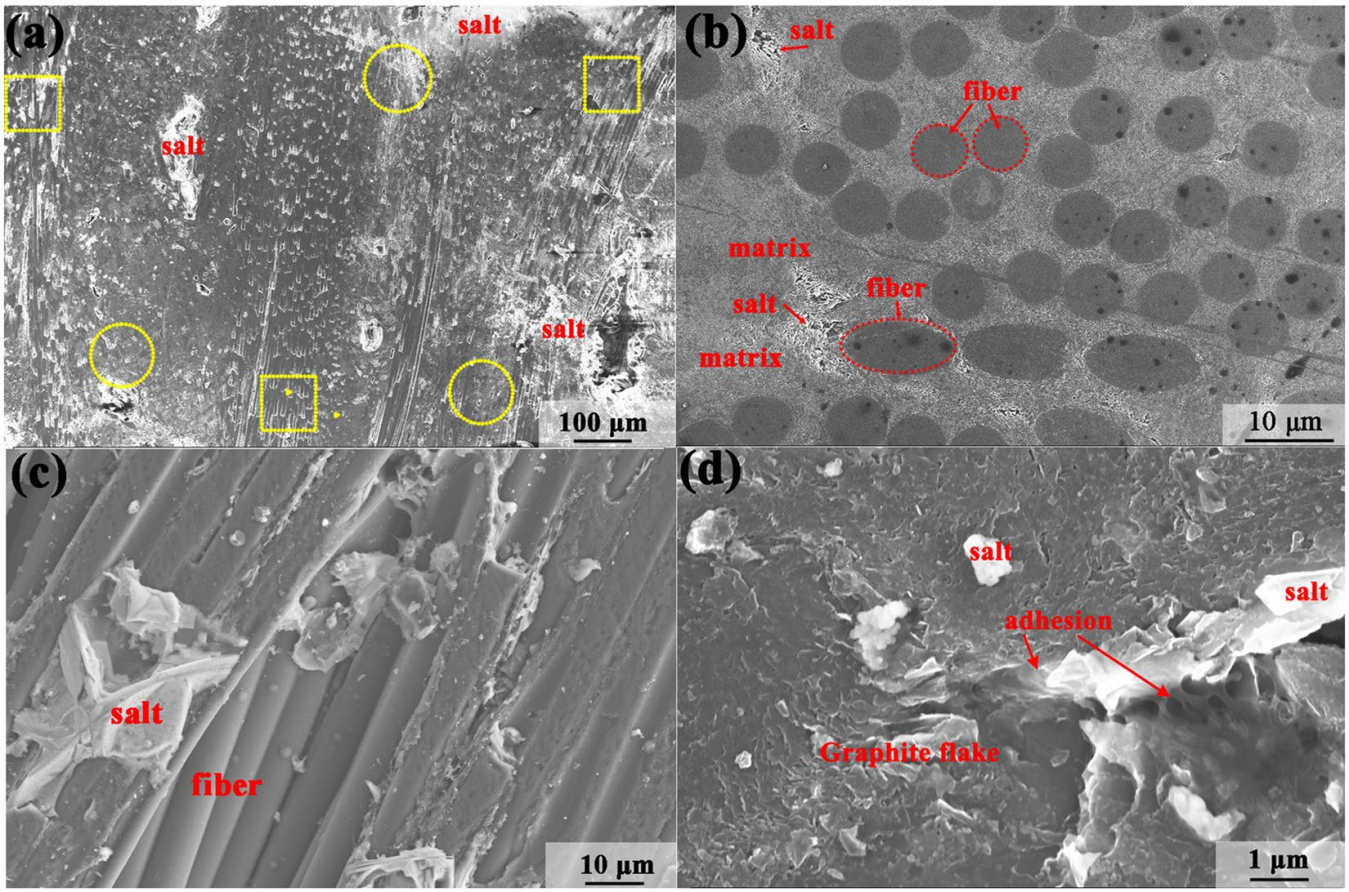
SEM images of C/C composite sample with FLiNaK salt impregnation. (**a**) The distribution FLiNaK salt (bright area) in the C/C composite sample; (**b**) and (**c**) represent the different regions marked with yellow circles and yellow box in Figure (**a**). (**d**) The salt dendrites are firmly fixed within the carbon matrix of C/C composite.

Figure 1 shows the morphologies of C/C composite sample. After FLiNaK salt impregnation experiment, FLiNaK salt (as labelled) can be clearly observed on the surface of the C/C composite sample. Figure 1a shows that the distribution of FLiNaK salt (bright area) in the C/C composite sample is homogeneous on the whole image. Figure 1b and c represent the different regions marked with yellow circles and yellow box in Fig. 1a. The C/C composite is composed of carbon fibers with an average diameter of around 6 μm and a carbon matrix. Figure 1a–c represent the carbon fibers and matrix in the C/C composite have an unordered distribution which make the C/C composite with low texture (as demonstrated by the results of 2D-GIXRD). The higher magnification SEM image of the sample (Fig. 1d) shows that the salt dendrites are firmly fixed within the carbon matrix of C/C composite.

### Synchrotron-based GIXRD analyses

**Figure 2:**
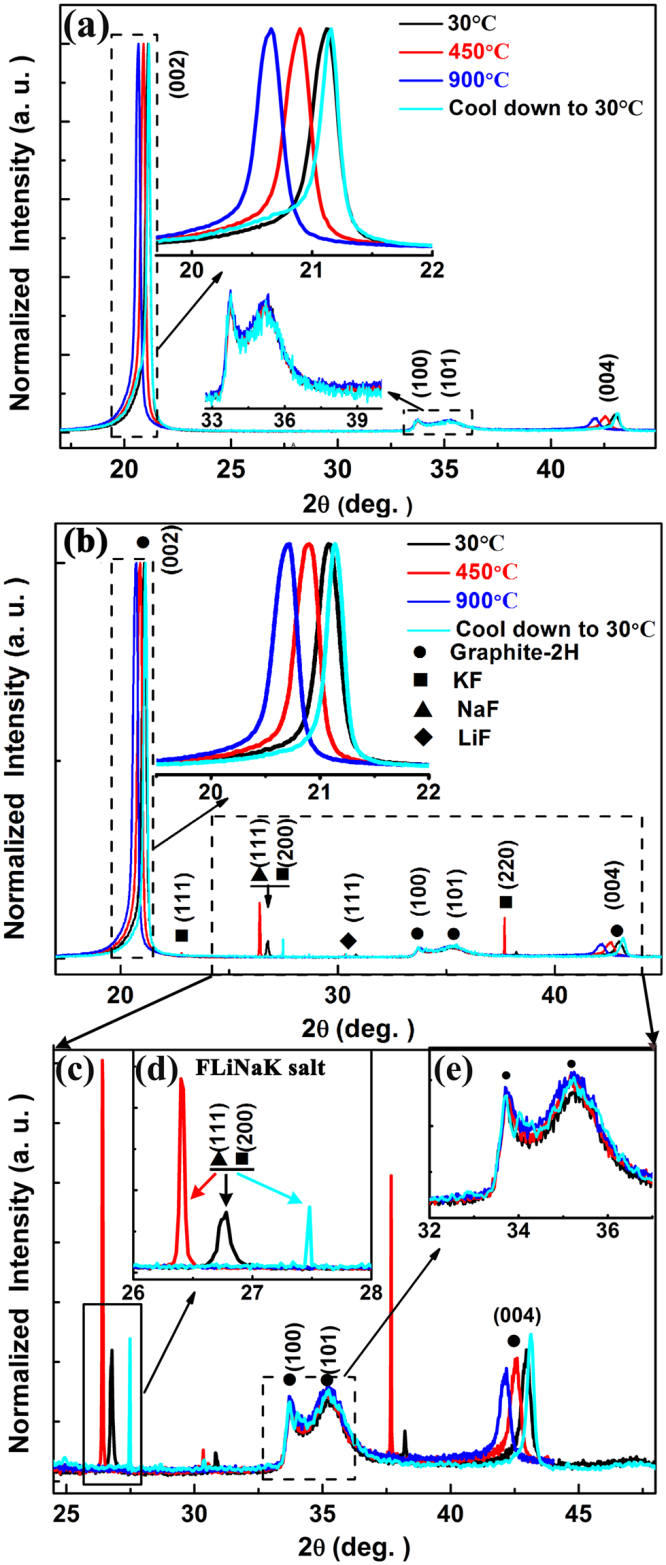
The *in-situ* synchrotron-based 1D-GIXRD patterns collected during the heating process of C/C composite sample without (**a**) and with (**b**) FLiNaK salt impregnation, (**c**) an enlargement of the dashed rectangle in (**b**), (**d**) the enlarged view of salt peaks, (**e**) the enlarged view of (100) and (101) peaks.

The *in-situ* 1D-GIXRD patterns collected during the heat-treatment process of C/C composite are displayed in Fig. 2. The patterns demonstrate that the graphite phase of C/C composite is mainly hexagonal (graphite-2H, space group: *P*63/*mmc*, JCPDS #: 41-1487)^27^. The fixed incidence angle (3°) not only ensures that X-ray beam penetrates deep enough below the surface to acquire an effective structure evolution information, but also keeps the sample relative stable in the crucible of heating device. For the virgin C/C composite as shown in Fig. 2a, with increasing temperature from 30 °C (RT) to 900 °C, the enlarged view of (002) peaks (inset of Fig. 2a) have presented a shift to the lower 2θ diffraction angle from 21.12° to 20.66°,(meanwhile the (004) peak, a senior diffraction peak, shows similarly shift trend), indicating that the interlayer spacing of the graphitic basal layers increased with temperature increase. While cooled down to 30 °C (RT), the (002) peak position of the virgin C/C composite shifts back toward higher diffraction angle than the original peak position (Fig. 2a), which interestingly demonstrates that the interlayer distance decreased to a smaller value after heat-treatment process. Similar evolution trend is also observed in the C/C composite with FLiNaK salt impregnation as shown in Fig. 2b. Particularly, the (002) peak position gradually shifts to the lower 2θ angle from 21.08° to 20.71° with the temperature increased from 30 °C to 900 °C, and returns to a higher diffraction angle 21.14° after cooled down to 30 °C (RT). For all of the specimens without and with FLiNaK salt impregnation (inset picture of Fig. 2a,e), it is notable that the (100) and (101) peak positions remain almost unchanged during heat-treatment process, implying that heat-treatment process and the impregnation of FLiNaK salt into the bulk of C/C composite do not affect the in-plane crystal structure of the graphite domains. Additionally, no position shift of (100) and (101) peaks could also provide an evidence that the position of the sample did not change during the whole heat-treatment process. These position evolutions of (002) diffraction peaks discussed above can be attributed to the micro-stress perpendicular to the c-axis while heating up and cooling down carbon materials^28–30^. It agrees well with the reports by Kelly^28^ and Li *et al*.^29^ that a tensile stress could affect the d-spacing of graphite (002) lattice planes during the heating and cooling process. However, the difference of (002) diffraction peaks position in the C/C composite with and without FLiNaK salt impregnation is necessarily related to salt. Thus, it is necessary to further investigate the crystalline behavior of FLiNaK salt in the graphite matrix of C/C composite during the heating and cooling process. As shown in Fig. 2b and c, the diffraction peaks of LiF (JCPDS #: 04-0857), NaF (JCPDS #: 36-1455) and KF (JCPDS #: 36-1458) were obviously observed for the C/C composite sample with FLiNaK salt impregnation. Figure 2c clearly shows that the peak position of FLiNaK salt shifts to lower 2θ angle during the heating process and then moves back to higher 2θ angle after cooled down treatment, which mean the lattice constants of FLiNaK salt increase during the heating process and following decrease after cooled down treatment. Particularly, the full width half maximum (FWHM) of FLiNaK salt diffraction peaks (Fig. 2d) gradually decrease with temperature increase from 30 °C to 450 °C. According to Scherer’s equation^31, 32^, the crystalline size of FLiNaK salt is estimated to be increased about one times at 450 °C than that at 30 °C. When the temperature is higher than melting point (454.0 ± 0.2 °C) of the FLiNaK salt, there is no salt diffraction peak showed in Fig. 2b at the temperature 900 °C due to the FLiNaK salt almost melting completely. After the specimens cooled down to 30 °C(RT), these diffraction peaks of LiF, NaF and KF reemerge and show a more narrow FWHM, indicating that the recrystallization of FLiNaK salt occurs with much higher crystallization. Notably, the crystallite size of FLiNaK salt is estimated to be increased about 2.8 times than that of before heat-treatment.

**Figure 3:**
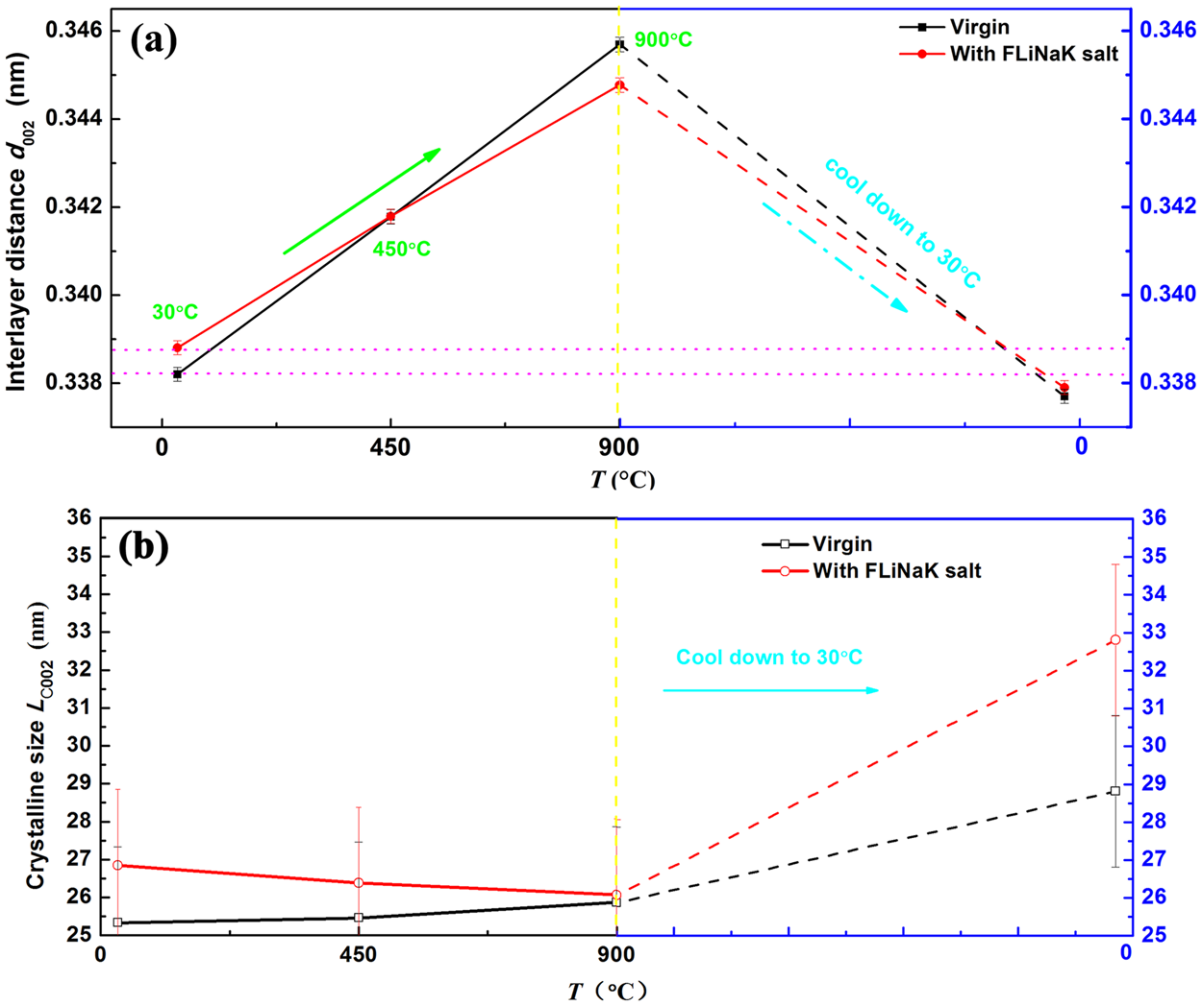
The *d*_002_ spacing (**a**) and crystallite size *L*_C002_ (**b**) at different temperatures during the heat-treatment process of C/C composite with and without FLiNaK salt impregnation.

Calculated by Bragg diffraction equation^32^ and Scherrer equation^32^, Fig. 3 shows the interlayer spacing *d*_002_ (Fig. 3a) and crystallite size *L*_C002_ (Fig. 3b) during the heat-treatment process of C/C composite with and without FLiNaK salt impregnation. In comparison to the conventional X-ray diffraction facilities, synchrotron-based X-ray diffraction could achieve a higher quality powder diffraction patterns in terms of peak profile shape and FWHM resolution^33^, which enables better resolving capability as well as fitting results for both qualitative and quantitative measurements^32^. Therefore, the errors of *d*_002_ and *L*_C002_ are very small and generally ignored in many related reports^33–35^. According to the previously reports^33, 34^, the error of *d*_002_ is estimated to be only about 10^−4^ nm order magnitude. Meanwhile, the error of *L*_C002_ is estimated to be about ±2 nm. Each error bar has been added in Fig. 3. As shown in Fig. 3a, the interlayer spacing *d*_002_ of all the samples with and without FLiNaK salt impregnation increases linearly with temperature during the temperature rising process, which mainly attributes to the crystallographic thermal expansion behavior^36^. The average crystallographic thermal expansion coefficient of interlayer spacing in the C/C composite with FLiNaK salt impregnation is estimated to be (1.54 ± 0.02)×10^−5^ K^−1^, which is smaller than (1.94 ± 0.02) ×10^−5^ K^−1^ of the C/C composite without FLiNaK salt impregnation. Similar phenomenon was observed in the cooling process, where the C/C composite with FLiNaK salt exhibited smaller crystallographic contraction coefficient. The different crystallographic thermal expansion/contraction for two sets of sample attributes to the suppression from FLiNaK salt impregnated.

Figure 3b shows the evolution of crystallite size *L*_C002_ (the crystalline coherence length in the 002 direction) in heating and cooling process. For two sets of sample, there is almost no difference in crystal size *L*_C002_ during the heating process, indicating similar crystallization of C/C composite with and without FLiNaK salt impregnation there. When the temperature cooled back to 30 °C (RT), the *L*_C002_ increased to ~28.81 nm from initial value of 25.30 nm for virgin sample and ~32.81 nm from 26.07 nm for the C/C composite with FLiNaK salt impregnated, respectively. It is easy to understand that the heating treatment improves the crystallization of the virgin C/C composite due to the internal residual stress effectively released in the process^25^. Notably, for the C/C composite with FLiNaK salt impregnation, besides the effect from the release of internal residual stress similar to the virgin sample, the crystallite size of FLiNaK salt was greatly increased after the cooling process which indicates the crooked carbon layers can be squeezed into flat layers^29, 37^, leading to the crystallite size increased along c-axis direction of C/C composite. Obviously, the *L*_C002_ of C/C composite with FLiNaK salt impregnation is larger than the virgin one after cooled down to 30 °C (RT), indicating that FLiNaK salt impregnation could facilitate the crystallization of C/C composite after whole heat treatment process.

**Figure 4:**
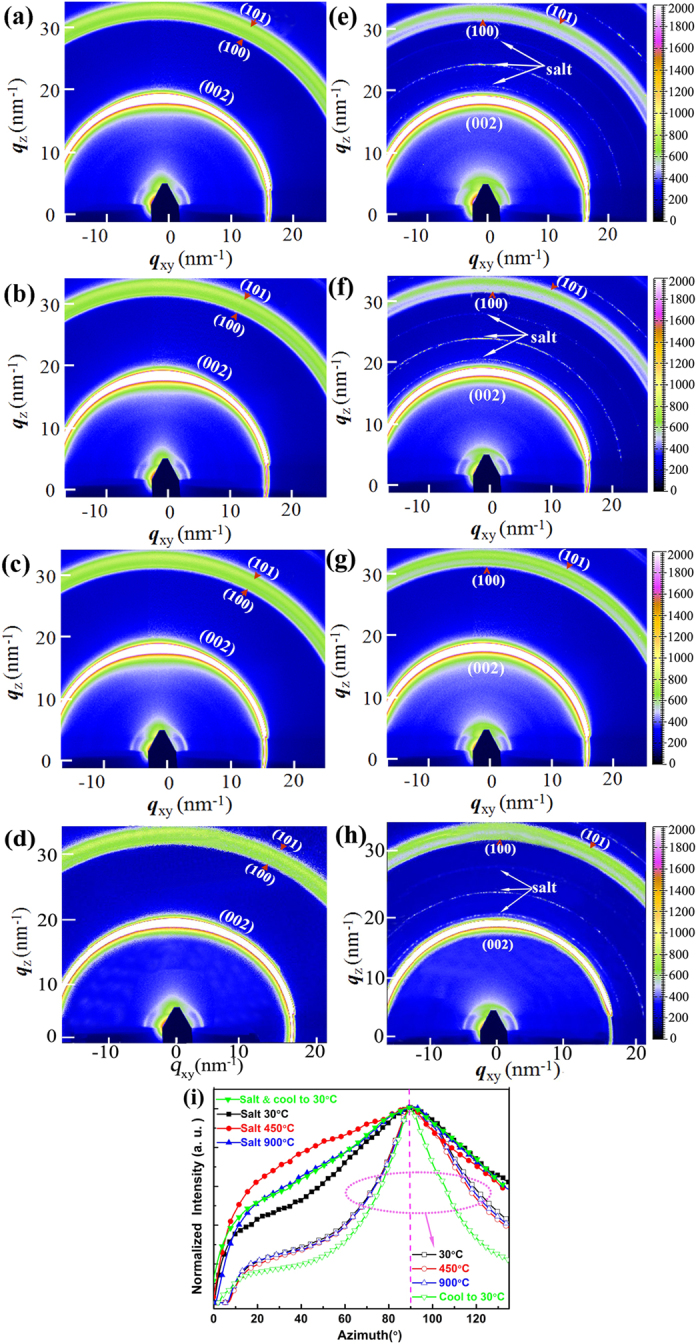
The *in-situ* synchrotron-based 2D-GIXRD patterns of C/C composite sample before (**a-d**) and after (**e-h**) FLiNaK salt impregnation. (**a**) and (**e**) Collected during the heating process at 30 ^o^C, (**b**) and (**f**) at 450 ^o^C, (**c**) and (**g**) at 900 ^o^C. (**d**) and (**h**) Cooled down to 30 ^o^C. (**i**) The corresponding radially integrated intensity plots along the ring at *q* ≈ 18.0 nm^−1^, assigned to the (002) plane of C/C composite.

However, 1D-GIXRD could only provide the limited structural information of the out-of-plane (OOP) diffraction patterns. In order to elaborately probe the crystallographic orientations of C/C composite with and without FLiNaK salt impregnation during the heat-treatment process, the *in-situ* real-time 2D-GIXRD diffraction combined with a fast 2D area detector based on a high brightness synchrotron X-ray source were further employed, which can provide more colorful information regarding the crystal texture and coarsening of C/C composite^36, 38–40^. Figure 4 shows the 2D-GIXRD profiles of C/C composite with and without FLiNaK salt impregnation at different heat-treatment temperature in a cell filled with the flowing N_2_. Extremely similar diffraction rings with a preferred orientation along the OOP direction were obtained, indicating highly textured crystal domains of C/C composite. The narrow and spotty scattered rings at *q* ≈ 20.6, 23.8, 27.4 nm^−1^ in Fig. 4e,f and h are from FLiNaK salt, which indicates that the molten salt with good crystallization has impregnated into the C/C composite sample after FLiNaK salt impregnation experiment. However, not any remarkable features at *q *≈ 20.6, 23.8, 27.4 nm^−1^ in Fig. 4g are observed as the FLiNaK salt in the C/C composites were almost completely melted at 900 °C.

It is well known that the crystallographic orientations of different structural domains in all directions can be examined in details by radially integrating the corresponding scattered ring^41^. In the following, the scattering ring corresponding to the typical (002) crystalline plane at *q *≈ 18.0 nm^−1^ of C/C composite was therefore radially integrated and plotted as functions of azimuth angle^41, 42^, as displayed in Fig. 4i. Less texture is observed for the crystal textures of C/C composites collected at different penetration depths achieved by changing grazing incidence angle of X-ray(cf. Figure S1), which further indicates the same crystal texture from surface to interior in the C/C composites. For the convenience of comparing the texture of these samples, the (002) crystalline diffraction intensities change with azimuth angle are normalized to the intensity at azimuth angle 90^o^, which could further reduce the influence of penetration depth in C/C composite materials with and without FLiNaK salt. It demonstrated clearly that the (002) planes in all the specimens have showed a preferential in-plane orientation leading to their sharpest peaks at an azimuth angle of 90^o^ in Fig. 4i. For the virgin specimen, the degree of orientation in the in-plane direction gets significantly more pronounced after the specimen cooled down to 30 ^o^C from higher temperature, which should be attributed to the release of internal residual stress in C/C composites after heat treatment^43, 44^. The preferential in-plane orientation of C/C composites with FLiNaK salt impregnation during the heating process becomes weaker than that of the virgin specimen, as indicated by the relatively broader peaks showed in Fig. 4i. In other words, crystallographic orientation in C/C composites with FLiNaK salt impregnation becomes random, which should be caused by the interaction between C/C composites and FLiNaK salt. The crystalline size of FLiNaK salt gradually increased with rising temperature lower than the melting point (454.0 ± 0.2 ^o^C), resulting in the weakened orientation order of C/C composite obviously showed in Fig. 4i. When the temperature is higher than the melting point (454.0 ± 0.2 ^o^C) of the FLiNaK salt, the prefer orientation order is improved due to the melting and outflowing of FLiNaK salt, which indicates the rigid contact effect from solidified FLiNaK salt on the texture of C/C composite. However, while the specimen cooled down to 30 ^o^C (RT) from higher temperature, it still keep the similar orientation order as that at 900 ^o^C due to the appropriate content of FLiNaK salt^20, 45^ left in C/C composite after the heat-treatment process since some FLiNaK salt flows out and aggregates at the bottom of the sample. From the analysis above, while the weight ratio of FLiNaK salt contented in C/C composite decreased, the orientation order of C/C composite was not markedly changed, in spite of the apparently increased crystallite size of FLiNaK salt after cooling process as analyzed in Fig. 2c.

As mentioned above, an appropriate content of FLiNaK salt impregnated into C/C composite could not only improved the crystallization of C/C composite, but also induced the weaken texture. The weight ratio of FLiNaK salt in C/C composite will influence the crystallization of C/C composite material and the texture change due to the complicated interaction between salt and carbon layers. The crystal structure evolution and micro-stress distribution in C/C composite induced by FLiNaK salt with weight change can be investigated in the future.

## Conclusion

In conclusion, the crystal structural evolution of C/C composite impregnated with FLiNaK salt during the heat-treatment process have been investigated by the *in-situ* real-time GIXRD. The average crystallographic thermal expansion rate of *d*_002_ in the C/C composite with FLiNaK salt impregnation ((1.54±0.02)×10^−5^ K^−1^) is smaller than that in the virgin sample ((1.94±0.02)×10^−5^ K^−1^). Similar phenomenon was observed in the cooling process, where the C/C composite with FLiNaK salt exhibited smaller crystallographic contraction rate. This thermal expansion/contraction difference attributes to the suppression of FLiNaK salt impregnated. The crystallite size *L*_C002_ of C/C composite with FLiNaK salt impregnation is larger than the virgin one after whole heat treatment process, indicating that FLiNaK salt impregnation could facilitate the crystallization of C/C composite after heat treatment process. Specifically, there is almost no difference in crystallite size *L*_C002_ for two sets of sample during the heating process, but there is an obvious increase after cooling process, indicating the synthetic action of the salt squeeze effect on crooked carbon layers and the release of internal residual stress. The texture distribution analysis of crystallographic orientations in C/C composite confirms that an appropriate content of FLiNaK salt impregnating into C/C composite could result in the change of graphite crystallite distribution in C/C composite and the weaken texture. These findings will promote to design the safer and more reliable C/C composite materials for the next generation molten salt reactor.

## Methods

### Sample preparation

The key parameters of C/C composite used in this study are shown in the supplementary material Table S1. The samples obtained from Aerospace research institute of materials & processing technology^46^ were fabricated using felt high modulus polyacrylonitrile (PAN)-based fiber^47^ preforms which were pressure-infiltrated and densified with molten pitch as matrix. In the composite, the volume fraction of the fiber is about 50%. The composite panels were sliced into pieces with size of 20 × 20 × 3 mm^3^. The pieces were marked by a laser marking machine, and then vacuum dried for 2 hours at 150 °C. To investigate the structure homogeneity of C/C composites before the impregnation experiments, the synchrotron-based XRD patterns for three randomly selected samples from one big bulk C/C composite are collected and shown in Figure S2. The diffraction and texture profiles resemble each other, which demonstrate that these samples exhibit similar crystal structure.

**Figure 5:**
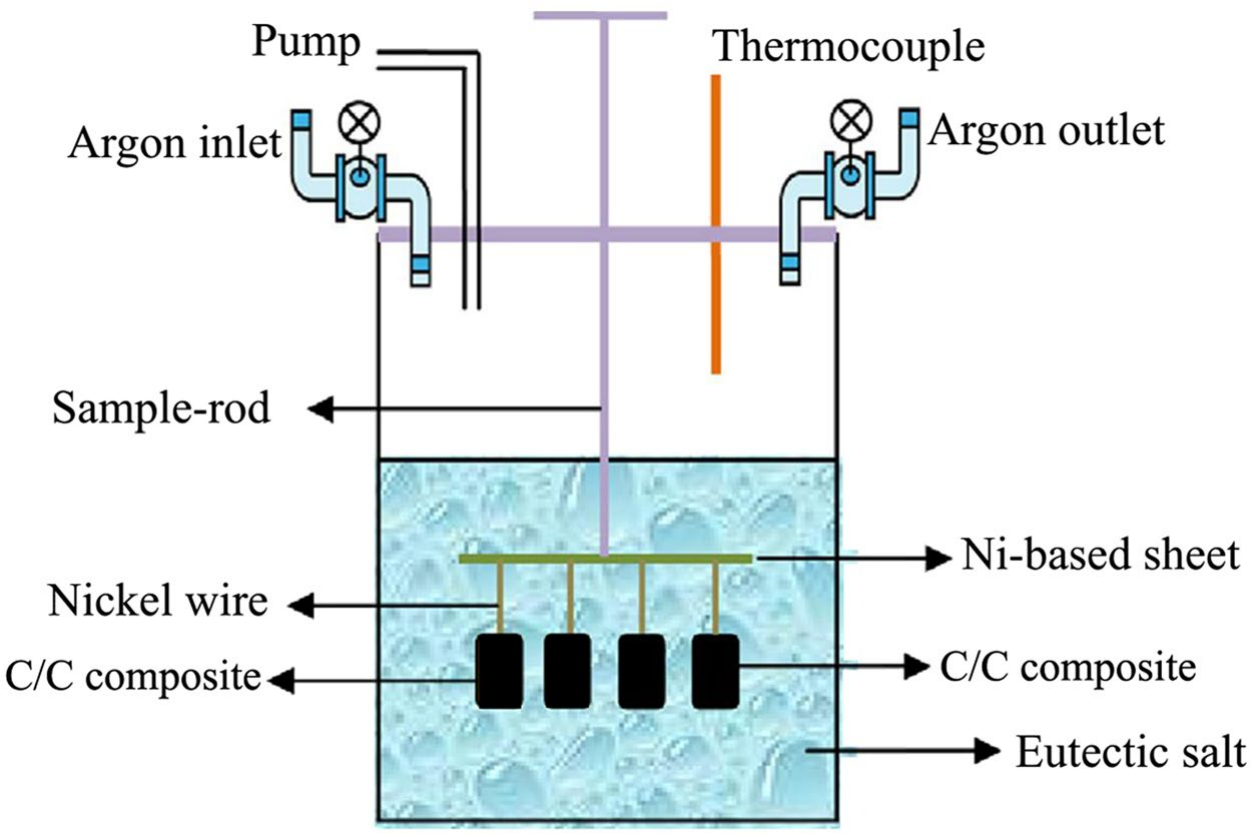
High pressure reactor used for molten salt permeation testing of C/C composite.

An eutectic composition was prepared using LiF (99.9% purity, from Aladdin Chemistry Co. Ltd.), NaF (99% purity, from Aladdin Chemistry Co. Ltd.), and KF (99% purity, from Acros Organics) salts mixed in proper proportions (46.5 mol% LiF/11.5 mol% NaF/42 mol% KF), exhibiting a melting point similar to that of the fluoride salt LiF–BeF_2_–ZrF_4_–ThF_4_–UF_4_ (70–23–5–1–1 mol%) used during the MSR experiment in Oak Ridge National Laboratory^48^. Henceforth this eutectic composition is denoted FLiNaK salt. The melting point of FLiNaK salt is measured precisely by Differential Scanning Calorimetry (DSC) technique and estimated to be 454.0 ± 0.2 ^o^C. The C/C composite samples and solid FLiNaK salt were introduced into a high-pressure reactor^49^ as shown in Fig. 5. A thermocouple was used to record the temperatures of the reactor. Argon was used as a protective gas and was also to pressurize the reactor. A sample rod supported a Ni based sheet to which the C/C composite samples were attached by nickel wires. After heating the reactor to 650 ^o^C to melt the salt, the C/C composite samples were immersed therein for 20 hours. The pressure of the reactor was kept at 5 atm. The FLiNaK salt contented in the impregnated C/C composite is calculated to be (10.1 ± 0.2) wt.% by weighting the mass change of C/C composite before and after FLiNaK salt impregnation experiment.

### Characterizations

**Figure 6:**
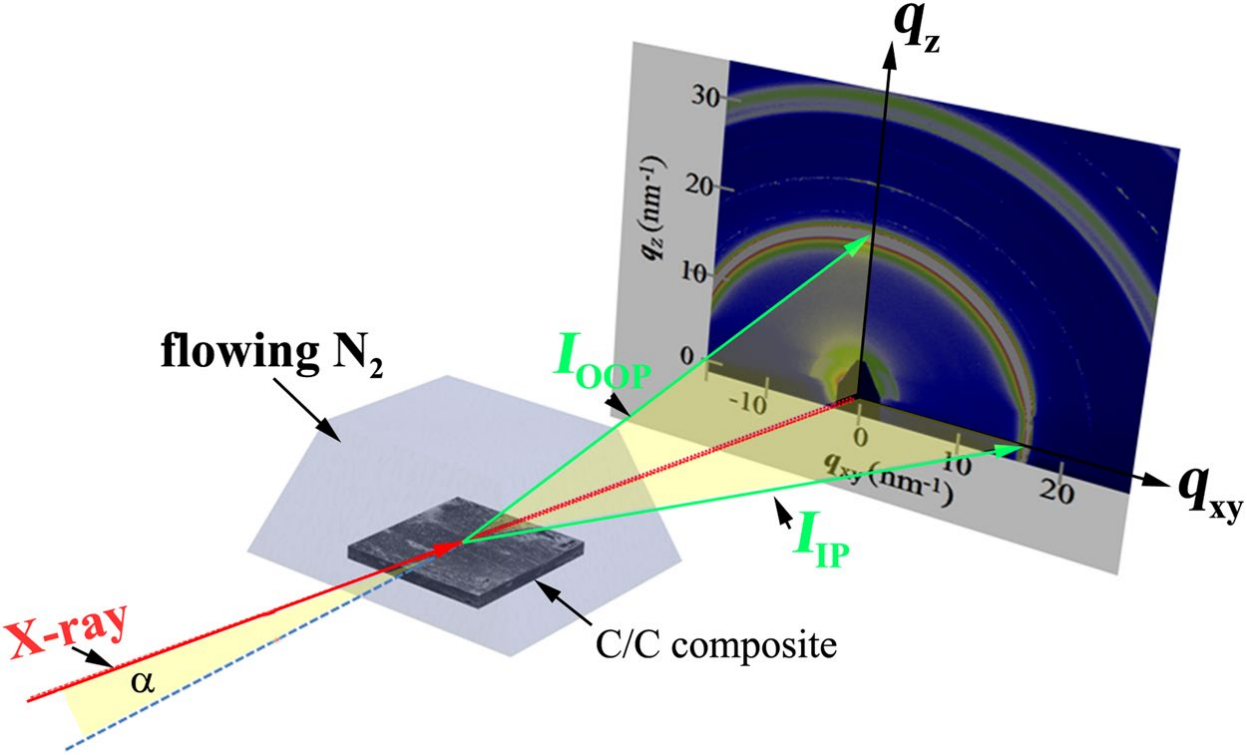
The GIXRD experimental setup, where *a*, *I*_OOP_ , *I*_IP_ , is the beam incident angle, the scattering beam in both out-of-plane (OOP) and in-plane (IP) directions, respectively.

SEM observations were carried out using a field-emission scanning electron microscope (LEO 1530 VP). The *in-situ* real-time GIXRD measurements during the heat-treatment process were performed at the BL14B1 beamline^50^ in Shanghai Synchrotron Radiation Facility (SSRF) at a wavelength of 0.124 nm^33, 51^. The beam was confined by vertical and horizontal slits and has a size of approximate 0.2 × 0.2 mm^2^ at the sample. The 1D-GIXRD patterns were obtained by using a NaI point detector with a scanning step of 0.02 degrees along the out-of-plane direction, and the grazing incidence angle of X-ray was 3° with respect to the surface plane. The 2D-GIXRD patterns were acquired by a MarCCD mounted vertically at a distance of around 229 mm from the sample with the exposure times less than 20 sec, and the grazing incidence angle of X-ray was also 3°. The 2D-GIXRD patterns were analyzed afterwards using the Fit2D software and displayed in scattering vector *q* (*q* = 4πsinθ/λ, where θ is half of the diffraction angle, and λ is the wavelength of incident X-ray). Figure 6 shows the GIXRD experimental setup, where *α*, *I*_*OOP*_, *I*_*IP*_, is the beam incident angle, the intensity of scattering beam along the out-of-plane (OOP) and in-plane (IP) directions, respectively. The sample was sealed in a cell filled with flowing nitrogen and heated to 450 ^o^C from room temperature (RT, 30 ^o^C), and kept at 450 ^o^C for 70 minutes. After that, the sample was continued to be heated to 900 ^o^C, and kept at 900 ^o^C for another 70 minutes to ensure the structural stability.

## Electronic supplementary material

Supporting Information
