# Supplementary material for: High temperature in-situ synchrotron-based XRD study on the crystal structure evolution of C/C composite impregnated by FLiNaK molten salt
Source: Sci Rep. 2017 Sep 6;7:10673. doi: 10.1038/s41598-017-11033-2 (PMC5587704; doi:10.1038/s41598-017-11033-2)
Supplement: Supplementary file 1 — Supporting Information [file 41598_2017_11033_MOESM1_ESM.doc]

Supporting Information

**High temperature *in-situ* synchrotron-based XRD study on the crystal structure evolution of C/C composite impregnated by FLiNaK molten salt**

Shanglei Feng ^*1, 2, 3,#^, Yingguo Yang^1, 2,3,#^, Li Li^1, 2,#^, Dongsheng Zhang^1^, Xinmei Yang^1^,, Huihao Xia^*1^, Long Yan^1^, Derek K. L. Tsang^1^, Ping Huai^1^, Xingtai Zhou ^*1^

^1^ *Shanghai Institute of Applied Physics, Chinese Academy of Sciences, 2019 Jialuo Road, Shanghai 201800, China*

^2^ *Shanghai Synchrotron Radiation Facility, Shanghai Institute of Applied Physics, Chinese Academy of Sciences, 239 Zhangheng Road, Shanghai 201204, China*

^3^ *University of Chinese Academy of Sciences*, *Beijing 100049*, *China*

**Table S1 The key parameters of C/C composite used in this study**

| **The key parameters of C/C composite** |  |
| --- | --- |
| Fiber/matrix type | PAN-based fiber /pitch-based matrix |
| Preform | Needled felt |
| Dimensionality | 2.5 |
| Flexural strength (MPa) | 120-160 |
| Density (g cm^−3^) | 1.8 |
| Fiber volume fraction (%) | 50 |
| Porosity (%) | 11±0.5 |


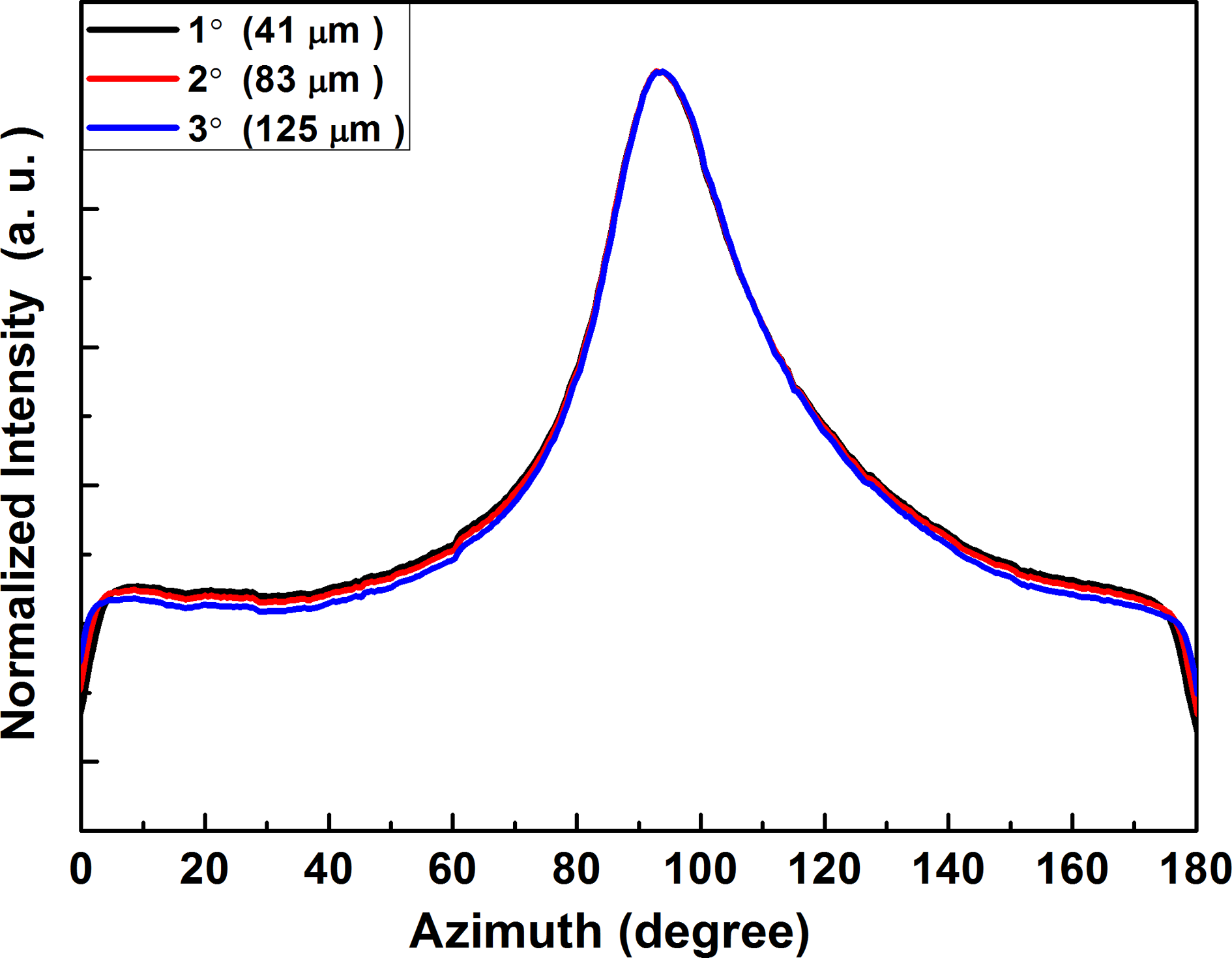


**Figure S1 Textures at different penetration depths. The (002) crystalline diffraction intensities change with azimuth angle are normalized to the intensity at azimuth angle 90°.**


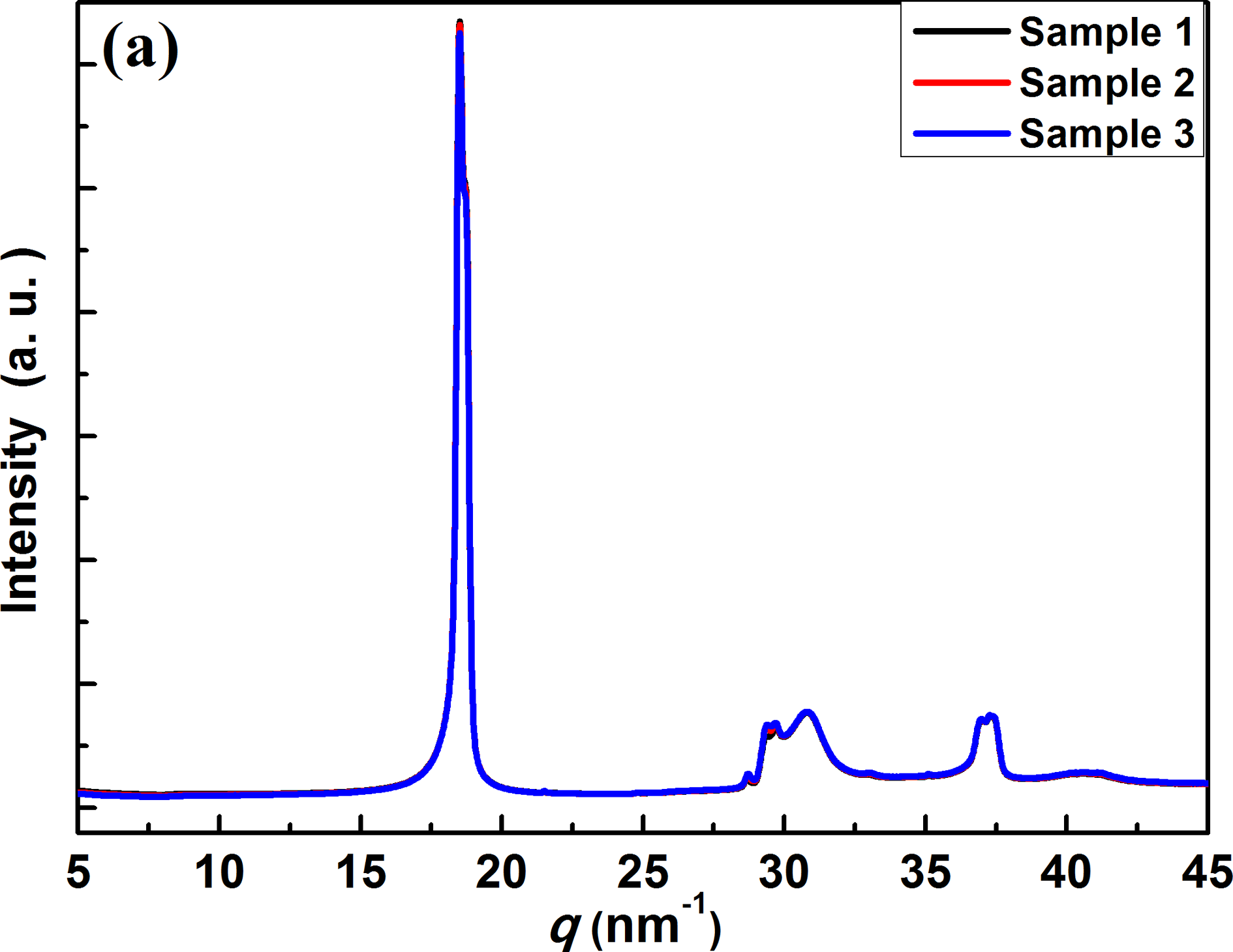

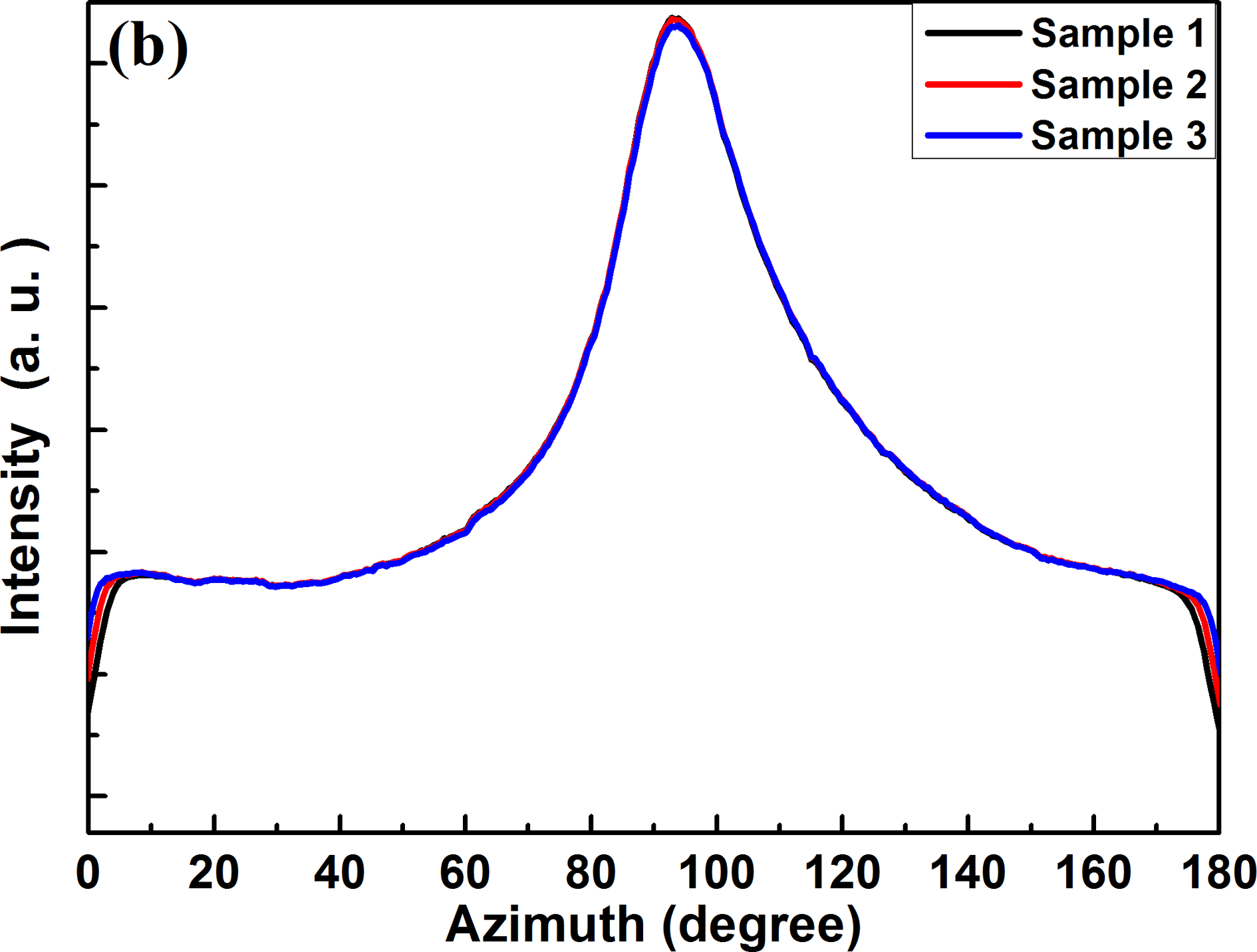


**Figure S2 (a) synchrotron-based XRD patterns collected from three randomly selected samples from one big bulk C/C composite without molten salt impregnation. (b) The corresponding radially integrated intensity plots along the ring at *q* ≈18 nm^-1^，assigned to the (002) plane of C/C composite.**
